# A longitudinal study of the characteristics and performances of medical students and graduates from the Arab countries

**DOI:** 10.1186/s12909-015-0482-3

**Published:** 2015-11-05

**Authors:** Ara Tekian, John Boulet

**Affiliations:** 1 Department of Medical Education, College of Medicine, University of Illinois at Chicago, 808 South Wood Street (MC 591), Chicago, IL 60612-7309 USA; 2 Foundation for Advancement of International Medical Education and Research, 3624 Market Street, Philadelphia, PA 19104 USA

**Keywords:** International medical graduates, Career pathways, International physician migration

## Abstract

**Background:**

While international physician migration has been studied extensively, more focused and regional explorations are not commonplace. In many Arab countries, medical education is conducted in English and students/graduates seek postgraduate opportunities in other countries such as the United States (US). Eligibility for residency training in the US requires certification by the Educational Commission for Foreign Medical Graduates (ECFMG). This study investigates ECFMG application trends, examination performance, and US physician practice data to quantify the abilities and examine the career pathways of Arab-trained physicians.

**Methods:**

Medical students and graduates from 15 Arab countries where English is the language of medical school instruction were studied. The performances (1^st^ attempt pass rates) of individuals on the United States Medical Licensing Examination Step 1, Step 2CK (clinical knowledge), and and a combination of Step 2CS (clinical skills) and ECFMG CSA (clinical skills assessment) were tallied and contrasted by country. Based on physician practice data, the contribution of Arab-trained physicians to the US healthcare workforce was explored. Descriptive statistics (means, frequencies) were used to summarize the collected data.

**Results:**

Between 1998 and 2012, there has been an increase in the number of Arab trained students/graduates seeking ECFMG certification. Examination performance varied considerably across countries, suggesting differences in the quality of medical education programs in the Eastern Mediterranean Region. Based on current US practice data, physicians from some Arab countries who seek postgraduate opportunities in the US are less likely to stay in the US following specialty training.

**Conclusion:**

Countries, or regions, with concerns about physician migration, physican performance, or the pedagogical quality of their training programs should conduct longitudinal research studies to help inform medical education policies.

## Background

The migration of physicians is a worldwide phenomenon [1]. Medical professionals emigrate for a number of reasons including lack of local resources, insufficient opportunities for graduate training, and a desire to improve their lives financially [2]. While the flow of physicans from one country to another is often characterized as “brain drain”, the lack of advanced practice training programs in some areas can induce individuals to seek opportunities elsewhere. While this travel can exacerbate workforce shortages in some areas, it may also foster educational and social improvements, provided that the emigrating physicians eventually return to their home country or provide some sort of reciprocal financial support.

Currently, approximately 25 % of the practicing physicians in the United States did not attend medical school in the the United States [3, 4]. These physicians, referred to as international medical graduates (IMGs), originate from many countries, including many Arab countries. While the contribution of IMGs to the US healthcare system has been documented in a number of studies, and it is clear that some regions (e.g., Caribbean) provide a large number of physicians to the US, more focused investigations of the contributions and characteristics of medical students and graduates from specific countries/medical schools are lacking [5–8].

To obtain a license to practice in the United States, IMGs must complete some postgraduate training (residency), normally at least 3 years. To enter a residency program, IMGs must be certified by the Educational Commission for Foreign Medical Graduates (ECFMG). The current requirements for ECFMG certification are listed elsewhere but include, amongst other criteria, primary source verification of the medical school diploma and successful performance on the United States Medical Licensing Examination (USMLE), which includes the Step 1 (Basic Science), Step 2CK (Clinical Knowledge) and Step 2CS (Clinical Skills) components [9]. For the most part, individuals who start the ECFMG certification process do so with the intent of obtaining residency training positions in the US, an essential requirement to obtain an unrestricted license to practice medicine in all US jurisdictions.

To better understand the various driving forces and possible reasons for seeking higher education and training in the US, it is important to study specific regions of the world, especially those where language issues are unlikely to hinder migration. Arab countries have interesting characteristics and conditions; medical education is conducted primarily in English, there is extreme wealth and poverty, government systems vary from dictatorship to full democracy, and there can be everything from political instability to relatively desirable living conditions. These qualities can certainly have some impact on immigration patterns, the quality of educational programs as measured through standardized examinations, and the individual motivation of international graduates to seek practice opportunities outside their home countries, particularly in the US. By exploring ECFMG application trends and certification examination performance, one can untangle some of the complexities of international medical education and provide a better description and understanding of the contribution of Arab-trained physicians to the US healthcare system. Such regional analytic studies will help policy makers, both in the US and abroad, to make informed policy decisions concerning medical education and immigration.

The certification process for IMGs can be quite challenging. Individuals must pay for and pass all examinations, one of which is only offered in the United States. It is not surprising that the performance of IMGs on these certification examinations has been quite varied, both over time and by country. Previous investigations indicate that, at least based on US medical licensing examination scores and outcomes, some countries, or medical schools within countries, produce more knowledgeable and skilled physicians [10, 11]. While individual performance on the examinations may fluctuate due to a number of factors (e.g., motivation, English language proficiency), aggregate performance (e.g., by medical school, country) may be more directly related to the quality of the educational and oversight (i.e., accreditation) program(s) [12]. It should be noted, however, that even in aggregate, performance on the ECFMG certification examinations is an imperfect marker of the quality of educational programs. The medical students and graduates who seek ECFMG certification may, or may not, be representative of all students/graduates from a particular program.

The purpose of this investigation is threefold: 1) to explore physician emigration trends from Arab countries to the United States; 2) to analyze and contrast examination performance for students/graduates who attended medical schools in Arab countries; and 3) to document the contribution of Arab-trained physicians to the US healthcare system.

## Methods

### Description of study population

For analysis of ECFMG application trends, the sample included all medical students and graduates from Arab countries where English is the language of instruction who applied for ECFMG certification in the period 1998-2012 (15 years). These countries include Bahrain, Egypt, Iraq, Jordan, Kuwait, Lebanon, Libya, Palestinian Territory, Oman, Qatar, Saudi Arabia, Sudan, Syria, United Arab Emirates, and Yemen. For the analysis of performance, 1^st^ attempt pass rates for all examinations (described below) for the 1998-2012 cohort were aggregated and compared. For the analysis of Arab contribution to the US workforce, the sample included all physicians who attended medical school in the countries noted above and were active in the US healthcare system (Administration, Full-time Hopital Staff, Residents, Locum Tenens, Medical Teaching, Office-Based Practice, Other and Research). It should be noted that Arab physicians who currently practice medicine in the US may not necessarily have applied for, or achieved, ECFMG certification between 1998 and 2012. This analysis of this data yields the current status of Arab-trained workforce in the United States, reflecting the emigration of Arab-trained physicians to the US over a much longer ECFMG certification window.

### Data sources

The number of medical schools in the study cohort of Arab countries was tallied from the International Medical Education (IMED) and Avicenna Directories [13, 14]. Although there are a number of Arab countries, such as Saudi Arabia, that may have more medical schools than the number reported in this manuscript, only the ones that were included in the IMED or Avicenna directories at the time of this investigation are part of this study; these medical schools are recognized by the World Health Organization (WHO) and/or are formally accepted by the Ministries of Higher Education and/or Health in the country where they are located.

ECFMG records were accessed to gather demographic information on the study cohort. The “application date” was coded at the date when the student/graduate first registered for an examination required for ECFMG certification. The year of this initial examination registration yielded the applicant year.

For the initial study period (1998-2012), examination performance data for all applicants was obtained from the National Board of Medical Examiners (NBME) via the ECFMG Master Database. The examination performance data included first-time pass rates for USMLE Step 1 (Basic Science), Step 2CK (Clinical Knowledge) and a combination of the ECFMG Clinical Skills Assessment (CSA) and USMLE Step 2CS (Clinical Skills). USMLE Step 1 (Basic Science) assesses whether the candidate can understand and can apply important concepts of sciences basic to the practice of medicine, with special emphasis on principles and mechanisms underlying health, disease, and modes of therapy. USMLE Step 2 CK (Clinical Knowledge) assesses whether the candidate can apply medical knowledge, skills, and understanding of clinical science essential for the provision of patient care under supervision and includes emphasis on health promotion and disease prevention. Both the ECFMG Clinical Skills Assessment (CSA), administered from 1998-2004, and USMLE Step 2 CS (Clinical Skills), administered from 2004 to the present, use standardized patients to evaluate medical students and graduates on their ability to gather information from patients, perform physical examinations, and communicate their findings to patients and colleagues.

The 2013 American Medical Association (AMA) Physician Masterfile was accessed to quantify the contribution of physicians trained in Arab countries to the US physician workforce. The AMA Masterfile lists all physicians practicing in the United States. As previously noted, these practicing physicians will not necessarily have applied for ECFMG certfication between 1998 and 2012; many would have applied before 1998.

To (1) study trends in physician emigration patterns, (2) document performance on national examinations of students who attended medical schools in Arab countries, and (3) quantify the contribution of Arab-trained physicians to the US health care system, we examined the following data from each Arab country: number of medical schools, population size, trends in ECFMG applicants, pass rates from US national licensure examinations (USMLE Steps 1 and 2), number of Arab-educated physicians practicing in the US physician workforce.

### Analysis

Descriptive statistics (means, frequencies) were used to summarize medical school numbers, ECFMG applications, certifications, ultimate certification rates, USMLE/CSA performance and number of practicing physicians in the United States.

Applicant data was based on all students/graduates who registered for an examination leading to ECFMG certification between 1998 and 2012 (15 year cohort). Since it can take a few years to achieve ECFMG certification (meet all the examination requirements), ultimate certification (meeting all the requirements for ECFMG certification, including passing all examinations) was only based on applicants in the 1998-2007 period. Ultimate certification was calculated as the percentage of 1998-2007 initial applicants who achieved ECFMG certification by September, 2013. For this analysis, individual applicants were tracked through the certification process.

To allow for valid comparisons between countries, performance data was based on first attempt pass rates. For clinical skills, both the ECFMG Clinical Skills Assessment and USMLE Step 2CS were combined.

The AMA Masterfile data (for those attending medical schools in Arab countries where the language of instruction was English) was summarized via frequencies and descriptive statistics. This study was approved by the Institutional Review Board of the University of Illinois at Chicago.

## Results

### Medical schools

The numbers of medical schools and population of the 15 Arab countries are presented in Table 1. Saudi Arabia (n = 34) has the greatest number of medical schools, followed by Sudan (n = 29). Overall, across all 15 countries, there is approximately 1 medical school for every 2 million inhabitants. While there are 19 medical schools in Egypt, this represents less than 1 medical school per 4 million inhabitants. The highest medical school density is located in Bahrain (3 medical schools for a population of 1.3 million).

**Table 1:** Number of medical schools and population size in 15 Arab countries

Medical School Country	Population (millions)	Number of medical schools	Medical schools/population (millions)
Bahrain	1.3	3	2.31
Egypt	82.6	19	0.23
Iraq	32.7	18	0.55
Jordan	6.6	4	0.91
Kuwait	2.8	1	0.36
Lebanon	4.3	7	1.63
Libya	6.4	11	1.72
Palestinian territory	4.2	3	0.71
Oman	3.0	2	0.67
Qatar	1.7	1	0.59
Saudi Arabia	27.9	34	1.22
Sudan	44.6	29	0.65
Syria	22.5	7	0.31
United Arab Emirates	7.9	5	0.63
Yemen	23.8	6	0.25
Total	272.3	150	0.55

### Emigration trends from the Arab countires

Table 2 shows the total number of initial applicants for ECFMG certification for the past 15 years by country of medical school and gender. Of the Arab countries included in this investigation, Egypt, Syria and Lebanon have had the most applicants, representing over 50 % of the total (n = 9093). Over all countries and the 15 year time period, 72.8 percent of the applicants were male.

**Table 2:** ECFMG applicants from 15 Arab countries with medical education taught in English, by gender (1998-2012)

Medical School Country	Male	Female	Total	Percent (of total applicants)
Bahrain	100	95	195	1.15
Egypt	3155	973	4128	24.29
Iraq	936	408	1344	7.90
Jordan	1476	437	1913	11.25
Kuwait	89	55	144	0.85
Lebanon	1603	790	2393	14.07
Libya	500	160	660	3.88
Palestinian Territory	84	27	111	0.65
Oman	67	52	119	0.70
Qatar	99	93	192	1.13
Saudi Arabia	1136	477	1613	9.48
Sudan	743	459	1202	7.14
Syria	2209	223	2572	15.12
United Arab Emirates	94	223	317	1.86
Yemen	76	11	87	0.51
Total	4623	12367	16990	100

Over the past 15 years (1998-2012) there has been some variability over time in the number of applicants from the Arab countries (Table 3). Beginning in 2000 (n = 527), there has been a relatively steady increase in the number of initial applicants, with a maximum being reached in 2011 (n = 2015). For some countries (data not shown), the growth in applicants has been quite remarkable. In the 5 years between 1998 and 2002 there were 144 applicants from Saudi Arabia. More recently (2008-2012), the number of initial applicants was 1194, an over 7-fold increase. The number fo female applicants has also been growing, going from a low of 121 in 2000 to a more recent high of 637 in 2012. In 2012, 1/3^rd^ of the initial applicants from this region were female.

**Table 3:** ECFMG applicants from 15 Arab countries with medical education in English, by application year and gender (1998-2012)

Application year	Male	Female	Total	% Female	Percent (of total applicants)
1998	791	253	1044	24.2	6.14
1999	419	135	554	24.4	3.26
2000	406	121	527	23.0	3.10
2001	475	119	594	20.0	3.49
2002	516	146	662	22.1	3.89
2003	469	166	635	26.1	3.73
2004	541	189	730	25.9	4.30
2005	667	248	915	27.1	5.39
2006	940	283	1223	23.1	7.20
2007	1001	356	1357	26.2	7.99
2008	963	356	1319	27.0	7.77
2009	1173	466	1639	28.4	9.65
2010	1292	551	1843	29.9	10.85
2011	1418	597	2015	29.6	11.84
2012	1296	637	1933	33.0	11.38
Total	12367	4623	16990	27.2	100

While initial applicants in this time period have come from a total of 117 (of 150) different medical schools in the 15 selected Arab countries, many were educated in just 5 institutions: University of Damascus Faculty of Medicine, Syria (n = 1543, 9.1 %); University of Cairo Faculty of Medicine, Egypt (n = 1184, 7.0 %); Ain Shams University Faculty of Medicine, Egypt (n = 1024, 6.0 %); Jordan University of Science and Technology Faculty of Medicine, Jordan (n = 992, 5.8 %); and American University of Beirut Faculty of Medicine, Lebanon (n = 896, 5.3 %).

### Performance

#### Ultimate certification

Of the 8248 students/ graduates who applied for ECFMG certification between 1998 and 2007 (inclusive), 5480 (66.4 %) achieved certification. Ultimate certification did, however, vary by country. Although based on only 33 people (registered for an initial examination between 1998 and 2007), 93.9 % of Qatari applicants achieved certification (as of September 2013). For the countries with the most applicants (Egypt, Syria, Lebanon) the ultimate certification rates were 54.7 %, 75.3 %, and 80.8 %, respectively. For all IMG applicants between 1998-2007 (n = 121,980), 65 % achieved ECFMG certification.

#### Examination performance

Examination performance of the study cohort is presented in Table 4. The data for each examination is based on first attempts taken between 1998 and 2012. For the Clinical Skills certification requirement, the first examination attempt may have been for the CSA (offered from 1998-2004) or USMLE Step 2CS (offered from 2004-2012).

**Table 4:** First attempt pass rates for USMLE Step 1, USMLE Step 2CK and CSA/USMLE Step 2CS

	USMLE Step 1-1^st^ attempt pass rate			USMLE Step 2CK-1^st^ attempt pass rate			USMLE Step 2CS/CSA – 1^st^ attempt pass rate		
Medical School Country	PASS	FAIL	%	PASS	FAIL	%	PASS	FAIL	P%
Bahrain	93	50	65.04	106	37	74.13	89	15	85.58
Egypt	2305	1103	67.64	2289	1365	62.64	1835	960	65.65
Iraq	917	300	75.35	866	242	78.15	782	229	77.35
Jordan	1388	138	90.96	1483	180	89.18	1243	317	79.68
Kuwait	54	60	47.37	66	43	60.55	48	13	78.69
Lebanon	2000	200	90.91	2018	145	93.30	1595	300	84.17
Libya	480	110	81.33	463	63	88.02	390	156	71.43
Palestinian Territory	71	4	94.67	94	6	94.00	52	26	66.67
Oman	34	37	47.89	60	22	73.17	15	9	62.50
Qatar	167	22	88.36	142	7	95.30	140	8	94.59
Saudi Arabia	863	403	68.17	844	280	75.09	814	264	75.51
Sudan	603	226	72.74	654	293	69.06	435	207	67.76
Syria	1938	316	85.98	1995	416	82.75	1507	582	72.14
United Arab Emirates	120	114	51.28	133	94	58.59	109	17	86.51
Yemen	37	21	63.79	39	28	58.21	28	13	68.30

Based on 1^st^ attempt pass rates for USMLE Step 1 (Basic Science), there was considerable variability in performance by country. Averaged over all schools within the country, both Oman and Kuwait had 1^st^ attempt pass rates of less than 50 %. On average, students educated in Jordan had the highest 1^st^ attempt pass rates (91 %). For all IMGs taking USMLE Step 1 for the first time between 1998 and 2012, the overall pass rate was 68.9 % (range by country: 47.4 % to 94.7 %). Eight of the 15 Arab countries studied had higher pass rates.

There was also country-based variability in 1^st^ attempt pass rates for Step 2CK. Here, on average, medical students and graduates from Qatar performed best (95 % 1^st^ attempt pass rate). Only the UAE and Yemen had 1^st^ attempt pass rates that were less than 60 %. For all IMGs taking USMLE Step 2CK for the first time between 1998 and 2012, the overall pass rate was 76.7 % (range by country: 58.2 % to 95.3 %). Seven of the 15 countries studied had higher pass rates.

There was somewhat less variability, across countries, on 1^st^ time performance on CSA/USMLE Step 2CS. Here, medical students and graduates from Qatar (94.6 % pass), the United Arab Emirates (85.5 % pass), Bahrain (85.6 % pass), and Lebanon (84.2 % pass) performed the best. For all IMGs taking CSA or USMLE Step 2CS for the first time between 1998 and 2012, the overall pass rate was 80.1 % (range by country: 62.5 % to 94.6 %). Four of the 15 countries studied had higher pass rates.

### Arab-trained physicians in the US workforce

Based on the 2013 AMA Masterfile, there are 891,413 active physicians (Administration, Full-time Hopital Staff, Residents, Locum Tenens, Medical Teaching, Office-Based Practice, Research, other) in the United States. Of these, nearly 1/3^rd^ (n = 291,759) were female. Only 1.6 % (n = 14,496) of the active physicians were educated in an Arab country where medical school instruction was in English. Within this Arab cohort, only 18.6 percent (n = 2702) were female.

Table 5 shows, by country, the total number of certificates issued by ECFMG from 1958 to 2012, the number of individuals in residency training, the total number of active physicians in the US and the approximate retention rate of Arab-trained physicians. The retention rate is approximate because a) not all ECFMG certficate holders obtain residency positions, b) some physicians entered the system before ECFMG certification requirements were manadatory, and c) some physicians, after residency training, will eventually return to their country of origin. For countries where at least 100 graduates obtained ECFMG certification, the approximate retention rates were highest for Syria (73.6 %) and lowest for Saudi Arabia (32.0 %).

**Table 5:** Contribution of Arab-educated physicians to the US workforce

Arab country	ECFMG Certficates Awarded (1958-2012)	Residents (Graduate Medical Education)	Number of active physicians in the US (includes residents)	Approximate US workforce retention rate (%)
Bahrain	108	29	50	46.3
Egypt	8138	528	4517	55.5
Iraq	2034	254	1171	57.6
Jordan	1892	424	1081	57.1
Kuwait	149	14	68	45.6
Lebanon	4329	551	2781	64.2
Libya	545	136	245	45.0
Palestinian Territory	56	20	27	48.2
Oman	26	1	2	7.7
Qatar	106	68	72	67.9
Saudi Arabia	959	199	307	32.0
Sudan	688	117	345	50.1
Syria	5121	565	3769	73.6
United Arab Emirates	103	28	45	43.7
Yemen	34	8	15	44.1
Total	24288	2942	14496	59.7

Note: Active physicans include administrators, full-time hopital staff, residents, locum tenens, medical teachers, office-based practitioners, other and clinical researchers

Based on Table 5, over 75 % of the Arab-trained physicians in the US were educated one of three countries: Egypt (n = 4517, 31.2 %); Syria (n = 3769, 26.0 %); Lebanon (n = 2781, 19.2 %). While all 15 countries provided physicians to the US workforce, over 50 % were educated in 1 of 5 medical schools: University of Damascus Faculty of Medicine (n = 2572, 17.8 %); American University of Beirut Faculty of Medicine (n = 1764, 12.2 %); University of Cairo Faculty of Medicine (n = 1617, 11.2 %); Ain Shams University Faulty of Medicine (n = 1349, 9.3 %); University of Alexandia Faculty of Medicine (n = 1008, 7.0 %).

## Discussion

While physicians trained in Arab countries do not represent a large percentage of ECFMG initial applicants, and currently make up a fairly small percentage of US physician workforce, they are a heterogenous group, both in terms of educational attainment and motivation to emigrate. Unlike other groups of physicians who seek educational opportunities outside of their country of medical school training, their characteristics and qualities have not been specifically studied [15, 16]. The motivation to emigrate is influenced by a number of factors such as higher income opportunities, the desire for greater stability and security, the perceived educational benefit from being able to access enhanced technology, and the lack of advanced graduate training locally [17, 18]. Given the sizeable growth of medical schools in this region, spurred by the demand for highly trained healthcare workers, it is important to know more about the educational programs in these countries and the relative abilities of the graduates.

Like many other parts of the world, there has been a growth in the number of medical education programs in the Arab countries. However, while the number of medical schools per given population is only a proxy for the adequacy of physician workforce supply, there is still great variability in the number of institutions per given population. Of particular note is Egypt where there are relatively few medical schools for the given population and a large number of physicians emigrating to the United States. The large physician density in Egypt (2.83 physicians/1000 inhabitants) would suggest sizeable medical school class sizes [19]. Given the challenges of education large classes, especially the provision of adequate clinical experiences, this may explain, at least partically, the moderate performance of this group on the examinations required for ECFMG certification, most notably the clinical skills component. Even if medical school class size does not have a major impact on the quality of instruction, the rapid expansion of medical schools, especially if trained faculty are scarce, is certain to put strains on some educational programs [20].

The number of IMGs taking the USMLE steps 1 and 2 exams has increased over the years. This trend, which is undoubtedly linked to the inherent motives of the test takers, is particularly important for IMGs coming from Arab countries. Globally, there is an international recognition of the USMLE step 1 and 2 examinations as markers of achievement or competence. For many medical school graduates, ECFMG certification provides the opportunity to apply to international residency training programs and eventually to practice in any part of the world. Successful candidates who get matched into US residency training programs benefit from the reputation and the prestige of being a graduate from a North American graduate medical education (GME) program, and consequently are highly regarded when they return back to their home countries and institutions. Most important, they can become the core faculty for expanding medical education programs.

In the Arab countries within the Eastern Mediterrenean Region, the quality of the undergraduate medical education training varies from one institution to another both within a country, and from one country to another. Our data, while based on a select cohort of individuals who take the examinations required for ECFMG certification, would suggest that quality of medical education (likely reflected in the curriculum, class size, length of training, clinical experience, etc.), and/or the selection of medical students, is superior in some Arab countries. For example, Lebanese medical students/graduates outperform Egyptian medical students/ graduates by at least 20 % (1^st^ time pass rates) on both Step 1 and Step 2CK. Although there are a number of explanations for the variability in performance across countries, the educational environment, financial resources, and the motivation of the individual to succeed cannot be discounted. A large number of medical graduates from Arab countries spend up to 18 months in the US preparing for the USMLE Step 1 and Step 2 examination [21]. There are many programs to prepare these Arab students to pass the USMLE exams [22]. While these programs can be costly, many countries from where the students originate provide financial aid to help these individuals pass these exams with the hope that they get admitted to US residency training programs. Although this information is not publicly available, some countries such as Saudi Arabia advertise the financial aid program through appropriate channels [23]. Success is important for the respective countries; they believe that such accredited programs produce high caliber graduates who in turn could specialize in any medical field and thus contribute to the improvement of the health care delivery system within their own countries. At a personal level, individuals are also quite motivated to complete US residency programs because, if they return to their home country, they are usually provided excellent positions with high paying salaries [24].

Medical students coming from well-resourced nations, such as the Gulf countries (Saudi Arabia, United Arab Emirates, Bahrain, and Oman), with the exception of Qatar, which caters to US citizens, have a great desire to return back home for the reasons mentioned above. Based on our longitudinal look at US workforce retention, albeit based on overlapping cross-sectional cohorts, only 32 % Saudi Arabian physicans who were certified between 1958 and 2012 were active in the US. This is likely an overestimate in that some of the active US physicans, namely residents and fellows, would eventually return to their home country. Medical students from relatively less well-resourced countries who complete their residency program in the US either attempt to stay in the US, or return to a richer Arab country to practice medicine. For example, nearly 75 % of Syrian physicians certified by ECFMG between 1958 and 2012 are currently active in the United States. It is clear that the migration of the physicians has political, economical and social implications [20]. While not specific to the Eastern Mediteranean Region, or the Arab world, physicans “brain drain” is characterized by emigration from poorer to richer nations, potentially exacerbating disparities in health care access.

Our investigation of the emigration and performance of medical students and graduates from Arab countries is not without limitations. First, the adequacy of the healthcare workforce, at least in terms of physicians, is only partially related to the number of educational institutions. Unfortunately, accurate longitudinal data concerning medical school graduation rates is difficult to obtain. Second, performance on ECFMG certification examinations (USMLE/ CSA) is an imperfect marker of the quality of medical education programs. Our data was based on all medical students/graduates who took examinations required for ECFMG certification. This cohort may or may not represent all students/ graduates from a given country. Finally, with respect to emigration, we only looked at inflow to the United States. Other countries such as Canada, the United Kingdom and Australia also provide training and practice opportunities for international medical graduates. A more complete picture of emigration from the Arab countries would necessarily demand data from other sources.

## Conclusion

The desire to improve the medical education system in the Arab countries is very high, and in some countries is the top priority for the Ministry of Higher Education and/or Health. One way to achieve that goal is to send graduates for advanced training to other countries such as the United States. While the value of this strategy depends on individuals returning to their home country, we do not know if this is an efficient way to improving the quality of medical education within a country. Moreover, findings from this study do not provide any information on how internationally-trained physicians practice in the United States or, for those who return, how they augment the local workforce and/or improve international medical education programs. As such, longitudinal research focusing on faculty development and healthcare outcomes, combined with a more detailed exploration of the determinants of performance on certification and licensing examinations, are topics for future study. Our data analyses, while specific to physicans educated in Arab countries, could easily be replicated for other countries, yielding multi-regional migration trends and more systematic, and standardized, comparisons of physician performance.
